# Lethal photosensitization of wound-associated microbes using indocyanine green and near-infrared light

**DOI:** 10.1186/1471-2180-8-111

**Published:** 2008-07-01

**Authors:** Ghada S Omar, Michael Wilson, Sean P Nair

**Affiliations:** 1 Division of Microbial Diseases, UCL Eastman Dental Institute, University College London, 256 Gray's Inn Road, London WC1X 8LD, UK; 2 Department of Surgery, Faculty of Physical Therapy, Cairo University, Cairo, Egypt

## Abstract

**Background:**

The increase in resistance to antibiotics among disease-causing bacteria necessitates the development of alternative antimicrobial approaches such as the use of light-activated antimicrobial agents (LAAAs). Light of an appropriate wavelength activates the LAAA to produce cytotoxic species which can then cause bacterial cell death via loss of membrane integrity, lipid peroxidation, the inactivation of essential enzymes, and/or exertion of mutagenic effects due to DNA modification. In this study, the effect of the LAAA indocyanine green excited with high or low intensity light (808 nm) from a near-infrared laser (NIR) on the viability of *Staphylococcus aureus*, *Streptococcus pyogenes *and *Pseudomonas aeruginosa *was investigated.

**Results:**

All species were susceptible to killing by the LAAA, the bactericidal effect being dependent on both the concentration of indocyanine green and the light dose. Indocyanine green photosensitization using both high (1.37 W cm^-2^) and low (0.048 W cm^-2^) intensity NIR laser light was able to achieve reductions of 5.6 log_10 _(>99.99%) and 6.8 log_10 _(>99.99%) in the viable counts of *Staph. aureus *and *Strep. pyogenes *(using starting concentrations of 10^6^–10^7 ^CFU ml^-1^). Kills of 99.99% were obtained for *P. aeruginosa *(initial concentration 10^8^–10^9 ^CFU ml^-1^) photosensitized by the high intensity light (1.37 W cm^-2^); while a kill of 80% was achieved using low intensity irradiation (0.07 W cm^-2^). The effects of L-tryptophan (a singlet oxygen scavenger) and deuterium oxide (as an enhancer of the life span of singlet oxygen) on the survival of *Staph. aureus *was also studied. L-tryptophan reduced the proportion of *Staph. aureus *killed; whereas deuterium oxide increased the proportion killed suggesting that singlet oxygen was involved in the killing of the bacteria.

**Conclusion:**

These findings imply that indocyanine green in combination with light from a near-infrared laser may be an effective means of eradicating bacteria from wounds and burns.

## Background

Wound infections are an ongoing problem not only for patients but also for healthcare providers worldwide. Infected wounds are responsible for significant morbidity and mortality, and an increase in the duration and the cost of hospital stay [1-3]. The growing resistance to conventional antibiotics among organisms that infect wounds and burns makes such infections difficult to treat [4-6]. Therefore there is a drive to develop novel antimicrobial strategies to which pathogens will not easily develop resistance.

One promising alternative is photodynamic therapy (PDT). Photodynamic therapy is the application of a non-toxic compound, termed a photosensitizer (PS) or light-activated antimicrobial agent (LAAA), which can be activated by light of an appropriate wavelength to produce reactive oxygen species (ROS) (i.e. singlet oxygen and free radicals) which can then exert a microbicidal effect [7,8]. Light of the appropriate wavelength excites the PS molecule into a triplet state which reacts with either a substrate to produce radical ions which in turn react with oxygen to produce cytotoxic species such as superoxide and hydroxyl radicals (type I reaction), or reacts directly with molecular oxygen to produce singlet oxygen (^1^O_2_) (type II reaction). PDT has a number of advantages over conventional antibiotics. Firstly, as the mechanism of killing is non-specific, with reactive oxygen species causing damage to many bacterial components, resistance is unlikely to develop from repeated use [9,10]. Secondly, both the PS and the light are applied locally to the target tissue; therefore reducing the risk of adverse systemic effects [11]. Cutaneous wound infections are particularly appropriate for treatment by PDT due to their easy accessibility to both a topical PS and light. The organisms most frequently responsible for infections of wounds and burns are *Streptococcus pyogenes*, staphylococci, such as methicillin-resistant *Staphylococcus aureus *(MRSA), and the Gram-negative bacterium *Pseudomonas aeruginosa *[1]. *Staphylococcus aureus *is a common wound-infecting organism which results in delayed epithelial closure of the wound, possibly due to its interaction with fibronectin and inhibition of keratinocyte migration [12]. The eradication of wound-infecting bacteria using lethal photosensitization has been reported in the literature, e.g. *Staph. aureus *[13-15], *P. aeruginosa *[16-19], *Strep. pyogenes *[20].

In this study we have investigated whether common wound-associated organisms are sensitive to lethal photosensitization using the dye indocyanine green (ICG) coupled with light from a near-infrared (NIR) laser emitting at 808 nm. This combination of PS and light source has a number of desirable characteristics over those described in the literature [13-20]. NIR laser light has a greater capacity to penetrate tissues than light of lower wavelengths whilst ICG is a NIR-absorbing water-soluble tricarbocyanine dye, which has been approved by the United States Food and Drug Administration (US FDA) for medical diagnostic studies. ICG has a very low toxicity and a high absorption at wavelengths around 800 nm. Recently, PDT with ICG has been used to treat tumors [21,22]. However it has, as yet, not been used for the PDT of infections with the exception of its topical use for the treatment of acne vulgaris [23-25].

## Methods

### Bacteria

Two Gram-positive organisms were used in this study; *Staph. aureus *strain 8325-4 and *Strep. pyogenes *strain 12202. In addition, the Gram-negative bacterium *Pseudomonas aeruginosa *strain PA01 was used. Gram-positive bacteria were maintained by weekly subculture on blood agar base (Oxoid Ltd, UK) supplemented with 5% horse blood whereas *P. aeruginosa *was subcultured on nutrient agar (Oxoid). *Staph. aureus *and *P. aeruginosa *were grown aerobically in nutrient broth (Oxoid) with shaking at 37°C for 16 h. *Strep. pyogenes *was grown in brain heart infusion broth (Oxoid) for 16 h at 37°C in an atmosphere of 5% CO_2_/95% air. Cells were then harvested by centrifugation and resuspended in phosphate buffered saline (PBS) to an optical density of 0.05 at 600 nm which corresponded to approximately 10^6^–10^7 ^colony forming units per ml (CFU ml^-1^) for the Gram-positive bacteria and 10^8^–10^9 ^CFU ml^-1 ^for *P. aeruginosa*.

### Photosensitizer

4,5-benzoindotricarbocyanine (Indocyanine green) C_43_H_47_N_2_NaO_6_S_2 _is a negatively-charged polymethine dye and was obtained from Sigma-Aldrich-UK. Fresh stock solutions were prepared immediately prior to each experiment in either PBS or sterile distilled water (H_2_O) or deuterium oxide (D_2_O) and kept in the dark.

### Laser light

A 500 mW Gallium-aluminum-arsenide (Ga-Al-As) NIR-Laser (Thor International Ltd – UK) emitting light with a wavelength of 808 ± 5 nm was used for irradiation. For experimental purposes, the distance of the laser probe to the plate surface was adjusted to give fluence rates of 1.37, 0.07 or 0.048 W cm^-2 ^with an actual power output of 470, 225 and 150 mW respectively.

### Effect of photosensitizer concentration on lethal photosensitization

Aliquots (50 μl) of a suspension of *Staph. aureus, Strep. pyogenes *(containing approximately 10^6^–10^7 ^CFU ml^-1^) or *P. aeruginosa *(10^8^–10^9 ^CFU ml^-1^) in sterile PBS were transferred into a 96-well plate and an equal volume of ICG in PBS was added to each well to give final concentrations ranging from 25 μg ml^-1 ^to 250 μg ml^-1^. After addition of the ICG, the wells were left in the dark for 10 or 30 minutes (pre-irradiation time) and then exposed to a measured dose of high intensity laser light at a fluence rate of 1.37 W cm^-2^. In this system, an exposure of 5 minutes corresponded to a light dose of 411 J cm^-2^. Each experimental condition was tested in at least triplicate and each experiment was carried out on at least two occasions. The conditions tested were; 1) controls which contained neither ICG nor received irradiation (L- S-), 2) incubation with ICG in the dark (L- S+), 3) irradiation in the absence of ICG (L+ S-) and 4) the test which was irradiated in the presence of ICG (L+S+).

To enumerate the surviving bacteria, serial 10-fold dilutions were plated in duplicate either on blood agar (*Staph. aureus *and *Strep. pyogenes*) or nutrient agar (*P. aeruginosa*).

### Effect of low intensity laser light

To avoid any heating effect that may occur during high intensity irradiation, the experiments were repeated using a laser power output of 150 mW to irradiate the samples at a low fluence rate of 0.048 W cm^-2^. Aliquots (50 μl) of a suspension of *Staph. aureus *or *Strep. pyogenes *(containing approximately 10^6^–10^7 ^CFU ml^-1^) in sterile PBS were transferred into a 96-well plate and an equal volume of ICG in PBS was added to each well to give final concentration of 25 μg ml^-1^. After addition of the ICG, the wells were exposed to a measured dose of low intensity laser light for 15 or 30 minutes corresponding to light doses of 43 and 86 J cm^-2 ^respectively. Similar experiments were performed with *P. aeruginosa *except that the concentration of ICG was 200 μg ml^-1 ^and using a laser power output of 225 mW with a fluence rate of 0.07 W cm^-2 ^for 35 minutes which corresponded to a light dose of 147 J cm^-2^.

### Effect of laser light dose

The effect of laser light dose on bacterial killing was investigated by varying the exposure time whilst the distance from the light source remained constant. The bacterial suspensions were prepared as described above. A photosensitizer concentration of 25 μg ml^-1 ^was used for photosensitizing the Gram-positive organisms while 200 μg ml^-1 ^was used for *P. aeruginosa*. Survival was determined after 1, 3, and 5 minutes irradiation at a high fluence rate of 1.37 W cm^-2 ^which corresponded to energy doses of 28, 85 and 141 J and energy densities of 82, 247 and 411 J cm^-2 ^respectively.

### Effect of serum on the lethal photosensitization of *Staph. aureus*

In order to investigate the lethal photosensitization of *Staph. aureus *in an environment similar to that which would exist in a wound, lethal photosensitization experiments were performed in the presence of 50% horse serum (HS). *Staph. aureus *was suspended in HS (~10^7 ^CFU ml^-1^) and ICG (in PBS) added at concentrations of 25 or 200 μg ml^-1 ^(bacteria were not washed after incubation with serum). After addition of ICG, samples of 100 μl were irradiated at a fluence rate of 1.37 W cm^-2 ^and light dose of 411 J cm^-2^.

### Effect of an enhancer of singlet oxygen life span on lethal photosensitization

To determine if singlet oxygen was involved in the lethal photosensitization process, D_2_O (Sigma-Aldrich. UK) was used to extend the life span of any singlet oxygen generated by exposure of ICG to laser light. *Staph. aureus *was suspended in D_2_O or H_2_O (~2 × 10^7 ^CFU ml^-1^) and ICG added to give a final concentration of 25 μg ml^-1^. Samples of 100 μl in triplicate wells were exposed to laser light for 1 minute at a fluence rate of 1.37 W cm^-2^. Control wells were also prepared and either were not exposed to light but received ICG or did not receive ICG and were not exposed to light or did not receive ICG and were exposed to light.

### Effect of a free-radical and singlet oxygen scavenger on lethal photosensitization

*Staph. aureus *was suspended in PBS (~2 × 10^7 ^CFU ml^-1^) or different concentrations of L-tryptophan (10 μM-10 mM) dissolved in PBS and incubated at 37°C for 1 h to detect any bactericidal activity of the scavenger itself. No bactericidal effect of the scavenger alone was detected. Samples of 100 μl in triplicate wells containing *Staph. aureus *and 25 μg ml^-1 ^ICG (L+ S+) in H_2_O (control) or the scavenger at a concentration of 10 mM were exposed to laser light for 1 minute at a fluence rate of 1.37 W cm^-2^. Control wells containing *Staph. aureus *and 10 mM L-tryptophan were also prepared and either did not receive ICG and were not exposed to light (L-S-) or were not exposed to light but received ICG (L-S+) or did not receive ICG and were exposed to light (L+S-).

### Measurement of bacterial suspension temperature during lethal photosensitization and the effect of these temperatures on the viability of *Staph. aureus *and *P. aeruginosa*

100 μL of the bacterial suspension, with final concentrations of 25, 100 or 200 μg ml^-1 ^ICG, were exposed to a measured dose of high intensity laser light for 1, 3 or 5 minutes (L+S+) to determine the temperature elevation during lethal photosensitization. Three additional wells contained the microbial suspension plus PBS instead of the photosensitizer were exposed to the same light doses to determine the rise of temperature in the absence of ICG (L+S-). The temperatures of the L+S- and L+S+ aliquots were recorded immediately before and after irradiation using an immersion thermocouple probe connected to a Fluke 179 digital multimeter (Fluke. USA). The experiments were carried out in triplicate.

Aliquots of *Staph. aureus *either in sterile PBS or in HS (containing approximately 10^7 ^CFU ml^-1^) were added to an equal volume of ICG to give a final concentration of 25 or 200 μg ml^-1^, whereas *P. aeruginosa *suspension in PBS was added to ICG to give a final concentration of 200 μg ml^-1^. Controls were prepared by adding an equal volume of PBS instead of ICG. The *Staph. aureus *suspensions were incubated either at 40°C (suspension in PBS) or 50°C (suspension in 50% HS) whereas *P. aeruginosa *was incubated at 50°C. All aliquots were incubated for 10 minutes in the dark. The survivors were enumerated by viable counting.

## Statistics

All experiments were performed twice and at least in triplicate except for lethal photosensitization experiments in horse serum with 25 μg ml^-1 ^which were performed twice but in duplicate. Values are expressed as means ± standard deviation. Comparisons between means of groups were analyzed using the Mann-Whitney *U *test. *P *= 0.05 was considered statistically significant.

## Results

### Indocyanine green is an effective photosensitizer of wound-infecting organisms

**Figure 1:**
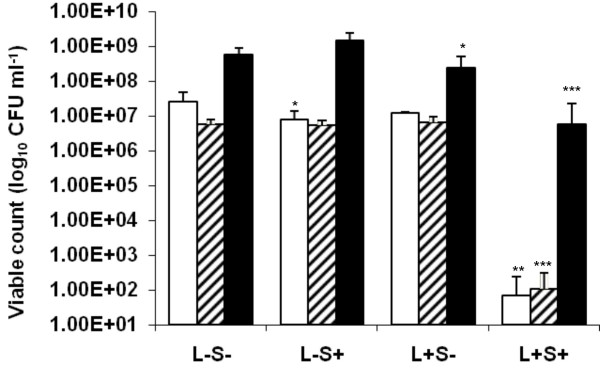
High intensity lethal photosensitization of *Staph. aureus* □ and *Strep. pyogenes* ▨ with 25 μg ml^-1 ^ICG or *P. aeruginosa* ■ with 200 μg ml^-1 ^ICG. An equal volume of either 50 μg ml^-1 ^(in the case of *Staph. aureus *and *Strep. pyogenes*) or 400 μg ml^-1 ^(in the case of *P. aeruginosa*) ICG (L-S+ and L+S+) or PBS (L-S- and L+S-) was added to each bacterial suspension, samples were left for 10 minutes in the dark and then irradiated at a fluence rate of 1.37 W cm^-2 ^with a light dose of 411 J cm^-2 ^from a NIR 808 nm laser (L+S- and L+S+) or kept in the dark (L-S+ and L-S-).

When *Staph. aureus *was treated with different concentrations of ICG and exposed to 411 J cm^-2 ^of high intensity NIR light a significant reduction in the viable count was achieved even with the minimum concentration of ICG used. For instance, when 25 μg ml^-1 ^of ICG was used there was a significant (*P *< 0.01) 5.56 log_10 _reduction in the viable count of the suspension which contained 2.55 × 10^7 ^CFU ml^-1 ^corresponds to 99.99% efficacy. Suspensions of *Staph. aureus *treated with ICG but not irradiated (L- S+) showed a small (0.5 log_10_) but significant (*P *< 0.05) reduction in the viable count (Figure 1).

When *Strep. pyogenes *was treated with different concentrations of ICG and exposed to 411 J cm^-2 ^of NIR light, a significant (*P *< 0.001) reduction in the viable count was achieved even with the lowest concentration of ICG. Treatment with 25 μg ml^-1 ^of ICG gave approximately a 4.7 log_10 _(99.99%) reduction in the viable count. This amounted to a kill of 5.66 × 10^6 ^CFU ml^-1^. Bacteria treated with ICG but not irradiated (L- S+), or those not treated with ICG but irradiated with NIR light did not show a significant reduction in viability (Figure 1).

When *P. aeruginosa *was treated with ICG, the concentration used to obtain a significant kill with the Gram-positive bacteria above was not sufficient to achieve killing upon irradiation. However, increasing the concentration of ICG to 200 μg ml^-1 ^gave a significant (*P *< 0.001) 2 log_10 _(99.1%) reduction in the viable count on irradiation. This equated to killing of approximately 5.99 × 10^8 ^CFU ml^-1^. Interestingly, irradiation of *P. aeruginosa *with NIR light in the absence of ICG resulted in a significant (*P *< 0.05) reduction in the viability of this organism (approximately 0.4 log_10 _reduction) albeit not as great as that achieved in the presence of ICG (Figure 1).

### The effect of various dye concentrations

**Figure 2:**
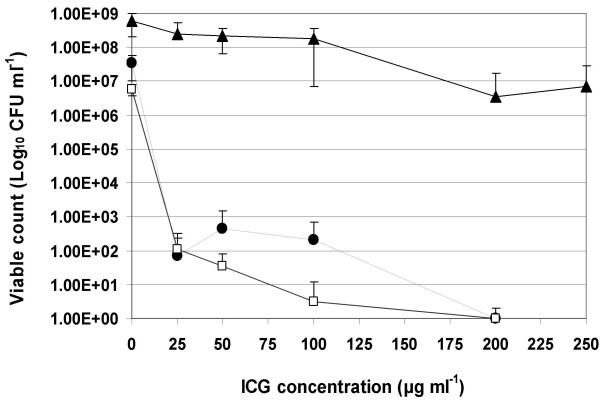
Lethal photosensitization of *Staph. aureus *●, *Strep. pyogenes *□ with 25,50, 100 and 200 μg ml^-1 ^ICG, and *P. aeruginosa *▲ with 25,50, 100, 200 and 250 μg ml^-1 ^ICG. An equal volume of the appropriate ICG concentration (L+S+) was added to each bacterial suspension, samples were left for 10 minutes in the dark and then irradiated at a fluence rate of 1.37 W cm^-2 ^with light dose of 411 J cm^-2 ^from a NIR 808 nm laser.

The bactericidal effect in all species was dependent on the ICG concentration. Figure 2 shows the log_10 _unit reduction in the viable count in the three micro-organisms when treated with different concentration of the PS and using a light dose of 411 J cm^-2 ^at a fluence rate of 1.37 W cm^-2^. The minimum effective concentration for killing the Gram-positive organisms was 25 μg ml^-1 ^whilst 200 μg ml^-1 ^was needed to kill *P. aeruginosa*.

### The effect of low intensity light

**Figure 3:**
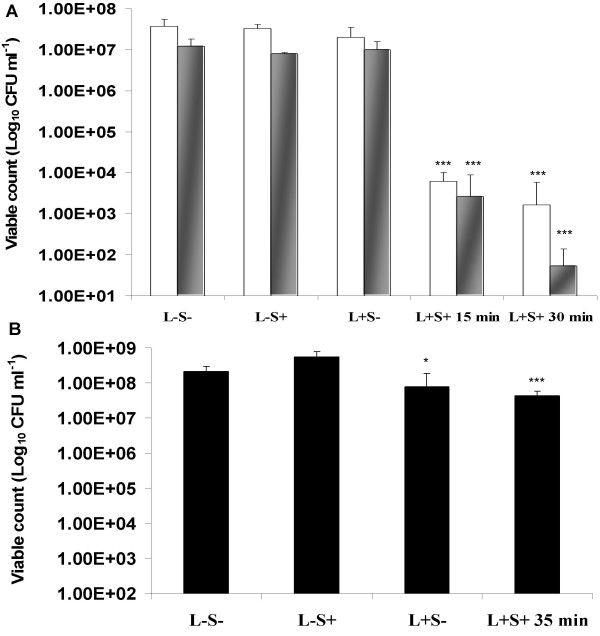
(A) Lethal photosensitization of *Staph. aureus *□ and *Strep. pyogenes * using a final concentration of 25 μg ml^-1 ^ICG. Samples were left for 10 minutes in the dark and then irradiated using a fluence rate of 0.048 W cm^-2 ^for 15 and 30 minutes, corresponding to light doses of 43 & 86 J cm^-2 ^respectively. (B) Lethal photosensitization of *P. aeruginosa *■ using a final concentration of 200 μg ml^-1 ^ICG. Samples were left for 10 minutes in the dark and then irradiated using a fluence rate of 0.07 W cm^-2 ^for 35 minutes, corresponding to light dose of 147 J cm^-2^. L-S- and L+S- were kept in the dark.

Irradiation of *Staph. aureus *in the presence of ICG for 15 or 30 minutes with a low fluence rate of 0.048 W cm^-2 ^achieved a significant (*P *< 0.001) reduction of 3.8 and 4.3 log_10 _in the viable count respectively (Figure 3A). Using the same conditions, irradiation of *Strep. pyogenes *resulted in significant (*P *< 0.001) reductions of 3.7 and 5.4 log_10 _in the viable count respectively (Figure 3A). For both gram positive-bacteria, kills amounting to 99.98 and 99.99% reductions in the viable count were achieved depending on the irradiation time. In the case of *P. aeruginosa*, a significant (*P *= 0.001) reduction of 0.7 log_10 _(80%) in the viable count was achieved in the presence of 200 μg ml^-1 ^ICG which was irradiated for 35 minutes at a fluence rate of 0.07 W cm^-2^, corresponding to a light dose of 147 J cm^-2^.

Amongst the three bacteria, only irradiation of *P. aeruginosa *with NIR light in the absence of ICG resulted in a significant (*P *< 0.05) 0.44 log_10 _(64%) reduction in the viability of this organism (Figure 3B).

### The effect of light dose

**Figure 4:**
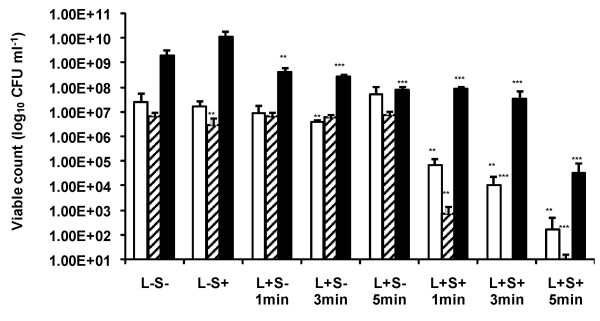
High intensity lethal photosensitization of *Staph. aureus* □, *Strep. pyogenes* ▨ with 25 μg ml^-1 ^ICG and *P. aeruginosa* ■ with 200 μg ml^-1 ^ICG. An equal volume of either 50 μg ml^-1 ^(in the case of *Staph. aureus *and *Strep. pyogenes*) or 400 μg ml^-1 ^(in the case of *P. aeruginosa*) ICG (L-S+ and L+S+) or PBS (L-S- and L+S-) was added to each bacterial suspension. Samples were left in the dark for 30 minutes and then (L+S- and L+S+) irradiated at a fluence rate of 1.37 W cm^-2 ^for 1, 3 or 5 minutes with light from a NIR 808 nm laser, corresponding to light doses of 82, 247, or 411 J cm^-2 ^respectively. Samples L-S+ and L-S- were kept in the dark.

During high intensity irradiation, the bactericidal effect was dependent on the light dose delivered. Significant (*P *< 0.01) reductions of 2.54 (99.7%), 3.36 (99.94%), and 5.15 log_10 _(99.99%) in the viable count of *Staph. aureus *were achieved using exposure times of 1, 3 and 5 minutes respectively. Significant (*P *< 0.001) log_10 _reductions in the viable count of *Strep. pyogenes *were 3.94 (99.99%), 6.82 (99.99%) and 6.13 (99.99%) using light doses of 1, 3 and 5 minutes respectively. In the case of *P. aeruginosa*, lethal photosensitization using exposure times of 1, 3 and 5 minutes achieved significant (*P *< 0.001) kills of 1.35, 1.75 and 4.74 log_10 _corresponding to 95.5, 98.2 and 99.99% reductions in the viable count respectively. However, in the absence of ICG, irradiation of *P. aeruginosa *also resulted in significant kills, with exposure times of 1, 3 and 5 minutes achieving log_10 _reduction in the viable count of 0.66 (78.2%), 0.85 (85.93%) and 1.36 (95.65%) respectively (Figure 4).

### Lethal photosensitization of *Staph. aureus *in the presence of serum

**Figure 5:**
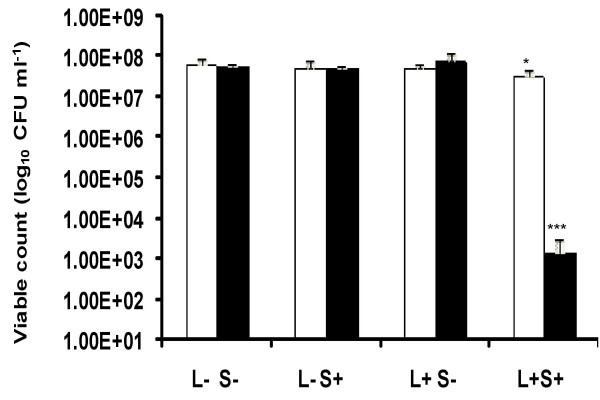
Lethal photosensitization of *Staph. aureus *in 50% horse serum (HS) using 25 □ or 200 ■ μg ml^-1 ^ICG. An equal volume of either 50 or 400 μg ml^-1 ^ICG (L-S+ and L+S+) or PBS (L-S- and L+S-) was added to *Staph. aureus *suspensions in HS and samples were left for 10 minutes in the dark and then irradiated at a fluence rate of 1.37 W cm^-2 ^and a light dose of 411 J cm^-2 ^using a NIR 808 nm laser (L+S- and L+S+) or kept in the dark (L-S+ and L-S-).

In the presence of HS, lethal photosensitization of *Staph. aureus *using a high fluence rate was significantly inhibited (Figure 5). Lethal photosensitization using 25 μg ml^-1 ^ICG and an irradiation time of 5 minutes in 50% HS gave a significant (*P *< 0.05) 0.3 log_10 _reduction in the viable count compared to a 5 log_10 _reduction in the absence of serum. However, increasing the concentration of ICG to 200 μg ml^-1 ^and exposure to the same light dose achieved a 4.61 log_10 _reduction in the viable count in the presence of serum (*P *< 0.001).

### Lethal photosensitization of *Staph. aureus *in the presence of a singlet oxygen scavenger or enhancer

**Figure 6:**
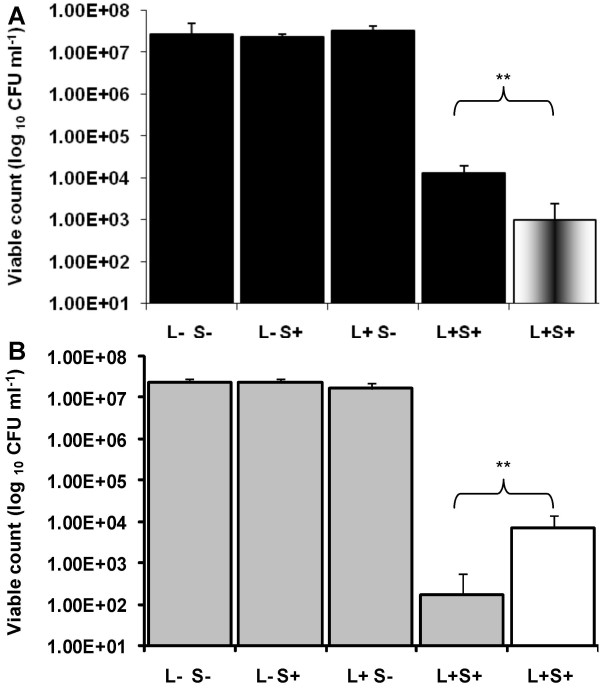
High intensity lethal photosensitization of *Staph. aureus *(A) suspended in 10 mM L-tryptophan ■ or suspended in H_2_O  and (B), suspended in D_2_O  or suspended in H_2_O □. In this experiment an ICG concentration of 25 μg ml^-1 ^was used and samples were pre-incubated in the dark for 10 minutes before being irradiated at a fluence rate of 1.37 W cm^-2 ^and light dose of 82 J cm^-2^.

The singlet oxygen scavenger, L-tryptophan, significantly reduced the lethal photosensitization of *Staph. aureus *(*P *< 0.01). One log_10 _reduction in the number of *Staph. aureus *killed was found in the presence of L-tryptophan over that in the absence of this scavenger (Figure 6A). Conversely, D_2_O considerably enhanced the killing of *Staph. aureus *with a 1.62 log_10 _greater reduction in viable counts compared to those achieved in the absence of D_2_O (*P *< 0.01) (Figure 6B).

### Temperature elevation during lethal photosensitization and its effect on the viability of bacteria

The temperature of the bacterial suspensions was elevated during high intensity lethal photosensitization with ICG. The rises in temperature depended on the light dose delivered and the concentration of the photosensitizer. The temperature of the bacterial suspensions increased from 22°C to 37°C during irradiation with the highest light dose and a dye concentration of 25 μg ml^-1^, whereas using 200 μg ml^-1 ^of ICG in either PBS or 50% HS, the temperature increased to a maximum of 47°C. No change in the viable count was observed after incubation of *Staph. aureus *suspended in PBS, in the absence or presence of 25 μg ml^-1 ^ICG, and incubated for 10 minutes at 40°C in the dark. Horse serum provided a protective effect for *Staph. aureus*, a slight increase or no change in the viable count was noted after *Staph. aureus *was suspended in PBS containing 50% HS in the absence or presence of 200 μg ml^-1 ^ICG and incubation for 10 minutes at 50°C. A statistically non-significant reduction was observed in the viable count of *P. aeruginosa *suspended in PBS, in the absence or presence of 200 μg ml^-1 ^ICG, and incubated for 10 minutes at 50°C. The viable count reduction was always less than 0.4 log_10 _(data not shown).

## Discussion

Indocyanine green is a water-soluble anionic photosensitizer which is widely used in medical diagnosis. It is approved by the US FDA for use in humans, in ophthalmology for retinal angiography as well as being used for diagnostic assessment of liver function and cardiac output and determining burn depth [26,27]. A number of *in vitro *and *in vivo *studies of the potential use of ICG-mediated PDT have been carried out. *In vitro *researches reported an inhibitory effect of photoactivated ICG on pancreatic cancer cells [28], colonic cancer cells [29], human (SKMEL 188) and mouse (S91) melanoma cells [30]. These studies suggested that ICG was a promising photosensitizer for clinical PDT but that further *in vivo *investigations were needed. Several authors have investigated ICG cyto-toxicity and photo-toxicity using *in vitro*, [31-34], *in vivo*, [35,36] and *ex vivo *models [37]. Some of these have reported phototoxicity in eukaryotic cells which was dependent on pre-irradiation time, ICG concentration, and light dose. Abels et al., 2000 reported that ICG phototoxicity depends on the pre-irradiation time [38]. After 1 h pre-irradiation time, no significant phototoxicity on HaCaT keratinocytes was detected using an ICG concentration as high as 50 μM and light doses of 48 J/cm^2 ^(80 mW/cm^2^). Phototoxicity of ICG at concentrations of 25 μM was detected after 4 h pre-irradiation while a concentration as low as 10 μM was phototoxic after 24 h [38]. However, in the current study the pre-irradiation time was no more than 30 minutes which implies that keratinocyte phototoxicity would not be a problem under these conditions.

In humans, ICG-mediated PDT has been used in the treatment of acne vulgaris [23-25]. A recent pilot study was carried out by Tuchin *et al*, (2003) on the effects of ICG photodynamic and photothermolysis treatment on acne vulgaris. In this study, the authors used a very high concentration of ICG (1.0 mg/ml), a pre-irradiation time of 5 or 15 minutes and very high light dose and fluence rates of NIR laser-diode light (803 or 809 nm). For soft acne treatment, the low intensity (803 nm, 10–50 mW/cm^2^, 5–10 minutes) or the medium-intensity (809 nm, 150–190 mW/cm^2^, 15 minutes) protocols were used. Single and multiple (up to 8–9) treatments were studied. The individual acne lesions were photothermally treated at 18 W/cm^2 ^(803 nm, 0.5 seconds) without skin surface cooling or at 200 W/cm^2 ^(809 nm, 0.5 seconds) with cooling. As no adverse effects were reported, the investigators concluded that such high light intensities and ICG concentrations were safe for use in humans [23].

The purpose of this study was to investigate whether ICG in combination with light from a NIR laser is effective at killing common wound-infecting organisms and to determine the fluence rates needed to photosensitize wound-infecting organisms. The results of the study have shown that all three organisms tested are susceptible to lethal photosensitization using ICG as a photosensitizer in combination with 808 nm light using both high and low fluence rates. At high fluence rates the Gram-negative bacterium *P. aeruginosa *showed no resistance to lethal photosensitization with ICG in the presence of high concentrations of the photosensitizer. However at the low fluence rate *P. aerusginosa *did show resistance to the lethal photosensitization process while the Gram-positive organisms (*Staph. aureus *and *Strep. pyogenes*) were not resistant. The lethal photosensitization of these wound-infecting organisms with ICG was dependent on the light dose and ICG concentration.

The differing susceptibilities of the Gram-negative and Gram-positive organisms to lethal photosensitization in this study are probably attributable to differences in cell wall structures. Gram-negative bacteria have an outer membrane that may reduce the uptake of reactive oxygen species by the bacterium [9]. In addition the presence of lipopolysaccharide (LPS) in the outer membrane of Gram-negative bacteria acts as a very effective permeability barrier to many of the molecules in the external environment and contributes to the development of drug-resistance in these organisms [39]. In contrast, Gram-positive bacteria have a porous outer layer of peptidoglycan which is a less effective permeability barrier [40]. Gram-negative bacteria are known to be relatively impermeable to neutral or anionic drugs and ICG is an anionic dye [41].

Interestingly, both high and low intensity 808 nm light alone was able to exert a cidal effect against *P. aeruginosa*. This killing effect was not heat mediated as the temperature of the bacterial suspensions did not exceed 33°C during irradiation. This finding is supported by the results of a previous study in which irradiation of the organism with 1–80 J cm^-2 ^laser light at a wavelength of 810 nm and using an irradiance rate of 0.03 W cm^-2 ^resulted in a significant inhibition of bacterial growth [42]. A possible explanation for this observation is that *P. aeruginosa *has endogenous pigments (pyoverdin and pyocyanin) that may absorb the light and result in the production of bactericidal species [16]. The current data suggest that NIR laser light irradiation by itself would also inhibit growth of *P. aeruginosa *in infected wounds.

A prerequisite for successful antimicrobial PDT to treat wound infection is its efficiency in the presence of wound fluid. To more closely mimic the conditions likely to be experienced *in vivo*, we investigated the effect of serum on the lethal photosensitization of *Staph. aureus*. Horse serum reduced the effectiveness of lethal photosensitization with ICG possibly because serum proteins in the environment bind to the ICG thereby preventing its uptake by the organism [43] or else they may act as quenchers of the singlet oxygen produced thereby protecting the bacterial cells from their lethal effects [44]. However, increasing the concentration of ICG overcame the inhibitory effect of serum suggesting that killing of the organism may be achievable *in vivo*. Our findings were confirmed by the results of Lambrechts et al. which showed that albumin inhibited the photodynamic inactivation (PDI) of *Staph. aureus *and exerted a dose dependent protective effect against the PDI of the microorganism which was dependent on the albumin content of the suspending fluid [45].

To reveal something of the underlying mechanisms involved in killing of the wound-infecting organisms by ICG, lethal photosensitization experiments were carried out in the presence of an enhancer of ^1^O_2 _life span (D_2_O) or a quencher of ^1^O_2 _(L-tryptophan) [46]. The augmentation of *Staph. aureus *killing in the presence of D_2_O demonstrated that enhancement of the life span of ^1^O_2 _increases the bactericidal effect thereby suggesting the involvement of this cytotoxic species in the lethal photosensitization process. Further evidence of the involvement of singlet oxygen is provided by the protective effect of the singlet oxygen scavenger, tryptophan.

However during the lethal photosensitization experiments at high fluence rate and the highest concentration of ICG (200 μg ml^-1^), the temperature of the bacterial suspensions increased to 47°C, whereas at lower concentrations the temperature did not exceed 42°C. According to the literature, temperatures of a few degrees above the physiological norm, i.e., 42–45°C can induce cell apoptosis. However, as normal tissues approach this temperature, intrinsic heat dissipation occurs (possibly due to augmented blood flow) so that temperatures up to 45°C could be tolerated by the tissues [47]. More prolonged heating can be associated with secondary necrosis of apoptotic cells, where the cells retained some of the features of apoptosis but had superimposed features of necrosis including membrane disintegration and swelling of cytoplasmic organelles. After a few days, apoptotic cells are phagocytosed by tissue mononuclear phagocytes. Temperatures greater than 45°C (45–48°C) cause prolonged necrosis, uniformly affecting all cells in tissue structure [48]. It also should be noted that heating has some synergic effect when applied together with PDT [49]; therefore more effective bacterial killing can be achieved. In clinical practice, providing an effective cooling system for the skin surface [50] or else the use of a pulsed laser may avoid tissue necrosis.

In order to ascertain whether this temperature rise was responsible for the kills achieved, the test organisms were exposed to a slightly higher temperature than that which occurred during lethal photosensitization and for longer periods of time. As exposure to these temperatures resulted in no, or only slight, reductions in bacterial viability, it can be concluded that the observed kills were attributable to the light-induced generation of singlet oxygen (and, possibly, free radicals) rather than to thermal effects. Even though the temperature of *Staph. aureus *suspensions containing 200 μg ml^-1 ^ICG increased up to 47°C, there was no significant reduction in the viable count of *Staph. aureus *in 50% HS at a similar temperature (50°C). It may be that protein molecules in the serum provide protection against the heating effect. This observation suggests that killing of *Staph. aureus *was more likely attributable to the products of lethal photosensitization. In addition, irradiating the bacteria with low fluence rates of 0.048 or 0.07 W cm^-2 ^resulted in significant reduction in the viability of the three bacteria. These results support the hypothesis that the cytotoxic species produced from the photosensitization process are responsible for bacterial kill.

The notion of disinfecting burns and wounds using a noninvasive and localized strategy such as PDT with limited damage to the host tissue is well documented in the literature [10,51,52]. Lambrechts et al achieved 3.6 or 4.8 log_10 _units reduction in the viability of *Staph. aureus *using 635 nm light with a light dose of 0.6 or 1.5 J cm^-2 ^and 1.56 μM 5-phenyl-10,15,20-tris(N-methyl-4-pyridyl) porphyrin chloride (PTMPP) [10]. Taking into account the variation in experimental design, we achieved a 5.5 log_10 _unit reduction in the viability of *Staph. aureus *using 25 μg ml^-1 ^ICG (32 μM) and a light dose of 411 J cm^-2 ^from a coherent light source (808 nm) which allows deeper tissue penetration than the light source (635 ± 15 nm) used in the above mentioned study. In another study, Orenstein et al. used a mixture of deuteroporphyrin and hemin which successfully disinfected burns infected with *Staph. aureus *even in the dark without illumination [15]. In contrast, the ICG used in the current study had minimal dark toxicity against the organisms tested. ICG is a comparatively weak photosensitizer because the yields of the triplet state appear low compared with other photosensitizers used in PDT [29]. Nevertheless, the data obtained in this study have shown that significant kills of *Staph. aureus *and *Strep. pyogenes *can be achieved using a low concentration of the photosensitizer of 25 μg ml^-1 ^and a low light dose of 43 J cm^-2^. The gram-negative organism, *P. aeruginosa*, appeared to be less susceptible as higher ICG concentrations and higher light doses were needed to achieve substantial kills. Interestingly, however, appreciable kills of the organism were attained by irradiation in the absence of the ICG. It is well established that humans can tolerate systemic ICG concentrations in the mg/ml range i.e. 5-fold greater than those used in this study.

The phototherapeutic window of many PSs effective against bacteria is 600–780 nm [53]. The most frequently studied PSs are activated by light wavelengths between 632.8–665 nm [54]. However, light of such wavelengths has limited tissue penetration compared to light with a wavelength of 808 nm [55] which we used to activate ICG in the present study. In fact, the depth of penetration doubles from 4 mm at 500–600 nm to 8 mm at 800 nm [56]. This may enable killing of bacteria within deeper wounds and burns. In addition, ICG is of very low toxicity and is rapidly excreted from the body so that any possible damage to host tissue would be limited.

## Conclusion

In summary, the results of the present study suggest that indocyanine green in combination with near-infrared light is a promising candidate for the photodynamic therapy of burn and wound infections. Furthermore, the NIR laser light itself may be able to disinfect wounds contaminated with *P. aeruginosa*. Use of this approach would reduce the requirements for systemic antibiotics in the management of skin infections and thereby help to reduce the emergence of antibiotic resistance. Although the results of these *in vitro *studies are promising, *in vivo *studies are needed to ascertain whether appreciable bacterial kills can be obtained in a wound model.

## Abbreviations

D2O: Deuterium Oxide; DNA: Diribonucleic acid; HS: Horse serum; ICG: Indocyanine green; LAAA: Light activated antimicrobial agent; NIR: Near Infra-red; PBS: Phosphate buffered saline; PDT: Photodynamic therapy; PS: Photosensitizer; ROS: Reactive oxygen species; US FDA: United State Food and Drug Administration.

## Authors' contributions

GSO: conceived of the study, participated in the study design, carried out the microbiological studies, performed the statistical analysis and drafted the manuscript. MW: conceived of the study, participated in its design and coordination and helped to draft the manuscript. SPN: conceived of the study, participated in its design and coordination, provided technical support and helped to draft the manuscript. All authors read and approved the final manuscript.
